# Ruddlesden–Popper Oxyfluorides La_2_Ni_1–*x*_Cu_*x*_O_3_F_2_ (0 ≤ *x* ≤ 1): Impact of the Ni/Cu Ratio on the Structure

**DOI:** 10.1021/acs.inorgchem.4c00399

**Published:** 2024-03-20

**Authors:** Jonas Jacobs, Hai-Chen Wang, Miguel A. L. Marques, Ke Xu, Jörn Schmedt auf der Günne, Stefan G. Ebbinghaus

**Affiliations:** † Faculty of Natural Sciences II, Institute of Chemistry, Inorganic Chemistry, Martin Luther University Halle-Wittenberg, Kurt-Mothes-Straße 2, Halle D-06120, Germany; ‡ Research Center Future Energy Materials and Systems of the University Alliance Ruhr, Faculty of Mechanical Engineering, Ruhr University Bochum, Universitätsstraße 150, Bochum D-44801, Germany; # Faculty IV: School of Science and Technology, Department of Chemistry and Biology, Inorganic Materials Chemistry, University of Siegen, Adolf-Reichwein-Str. 2, Siegen D-57076, Germany

## Abstract

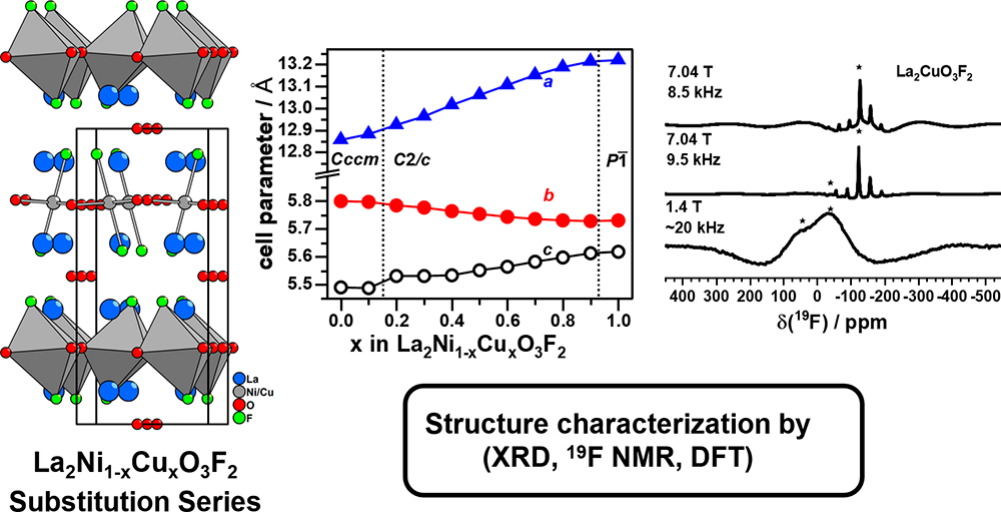

Ruddlesden–Popper oxyfluorides La_2_Ni_1–*x*_Cu_*x*_O_3_F_2_ (0 ≤ *x* ≤ 1) were obtained by topochemical reaction of oxide precursors La_2_Ni_1–*x*_Cu_*x*_O_4_, prepared by citrate-based soft chemistry synthesis, with polyvinylidene fluoride (PVDF) as the fluorine source. Systematic changes of the crystal structure in the oxide as well as the oxyfluoride substitution series were investigated. For 0.2 ≤ *x* ≤ 0.9, the oxyfluorides adopt the monoclinic (*C*2/*c*) structural distortion previously solved for the *x* = 0.8 compound based on neutron powder diffraction data, whereas the sample with a lower Cu content of *x* = 0.1 crystallizes in the orthorhombic (*Cccm*) structure variant of La_2_NiO_3_F_2_. The orthorhombic-to-monoclinic structural transition was found to be the result of an additional tilt component of the Jahn–Teller elongated CuO_4_F_2_ octahedra. The structural transitions were additionally studied by DFT calculations, confirming the monoclinic space group symmetry. The “channel-like” anionic ordering of the endmembers La_2_NiO_3_F_2_ and La_2_CuO_3_F_2_ was checked by ^19^F MAS NMR experiments and was found to persist throughout the entire substitution series.

## Abstract 2

Ruddlesden−Popper oxyfluorides La_2_Ni_1−*x*_Cu_*x*_O_3_F_2_ were successfully prepared, exhibiting strongly distorted variants of the K_2_NiF_4_ structure with “channel-like” oxygen interlayer occupation. The structural evolution was studied in increments of Δ*x* = 0.1 by XRD, ^19^F MAS NMR, and DFT calculations.

## Introduction

Ruddlesden–Popper (RP) oxides have emerged as a focal point of research in the field of advanced materials due to their intriguing structural properties and potential applications in areas such as energy storage,^1,2^ catalysis,^3,4^ and electronics.^5,6^ Ruddlesden–Popper compounds are characterized by a layered crystal structure (often written as (AX)(ABX_3_)_*n*_), which distinguishes them from traditional perovskites (ABX_3_). The arrangement of perovskite layers with thickness *n* alternating with one halite-type (AX) layer in the RP structures results in distinctive electronic, magnetic, and optical properties, making them interesting for both fundamental research and industrial applications. Ruddlesden–Popper cuprates A_2_CuO_4_ are highly prominent representatives for the *n* = 1 member (K_2_NiF_4_ structure type) of the series due to the first discovery of nonmetal superconductivity in La_2–*x*_Ba_*x*_CuO_4–*y*_ by Bednorz et al.^7^ Anionic substitution is widely used to obtain mixed ionic RP compounds with strongly altered structural distortions, which inflict the physical properties. One particular class of materials are the oxyfluorides, of which an increasing number of compounds with different O/F compositions are known to date. These oxyfluorides range from compounds with small fluoride excess like superconducting La_2_CuO_4_F_δ_ (0 < δ < 0.25)^8−10^ over stoichiometric compounds with O + F = 4 like Sr_2_FeO_3_F^11^ to highly fluorinated compounds with O + F > 4 (e.g., LaSrMnO_4_F_1_,^12^ La_2_CoO_4_F_1.2_,^2^ Ba_2_ZrO_3_F_2_,^13^ and Ba_2_SnO_2.5_F_3_^14^). Different low-temperature fluorination methods like the solvothermal reaction with XeF_2_^10^ as well as reactions with the fluorine-containing polymers polyvinylidene fluoride (PVDF)^15−17^ and polytetrafluoroethylene (PTFE)^18^ have emerged in recent years, yielding less oxidative synthesis conditions compared to the reaction with F_2_ gas, enabling the synthesis of less redox stable oxyfluorides.

Lately, oxyfluorides La_2_NiO_3_F_2_,^19^ La_2_Ni_0.2_Cu_0.8_O_3_F_2_, and La_2_CuO_3_F_2_^20^ were successfully synthesized by the topochemical fluorination of corresponding oxides with PVDF. These compounds share an unusual structural distortion scheme with of strong octahedral tiltings, which result in the formation of a channel-like arrangement of the interstitial anionic positions within the AX layer.

For oxides La_2_NiO_4_–La_2_CuO_4_, the whole solid solution series has been described.^21,22^ In this study, we report on the successful synthesis of the corresponding oxyfluorides La_2_Ni_1–*x*_Cu_*x*_O_3_F_2_ (0.0 ≤ *x* ≤ 1.0 with Δ*x* = 0.1). The samples were obtained through topochemical low-temperature fluorination of reactive oxide precursors prepared by the citrate route with PVDF as a fluorination agent. All oxyfluorides were found to adopt the distorted K_2_NiF_4_ structure variant found for La_2_NiO_3_F_2_ with a transition from orthorhombic (*Cccm*) to monoclinic (*C*2/*c*) symmetry for 0.2 ≤ *x* ≤ 0.9 due to the Jahn–Teller active Cu^2+^. The stability of both space groups was investigated by DFT calculations, verifying the structural transition. Additional ^19^F MAS NMR experiments were applied to check the proposed anion ordering of both endmembers (i.e. *x* = 0 and 1) as well as two of the Ni/Cu-substituted compounds (i.e. *x* = 0.3 and 0.7).

## Experimental Section

### Synthesis

Precursor oxides La_2_Ni_1–*x*_Cu_*x*_O_4_ were synthesized in steps of *x* = 0.1 by a citric acid-assisted combustion method.^23^ Stoichiometric amounts of La_2_O_3_ (Merck) (dried at 900 °C for 10 h), Ni powder (Sigma-Aldrich), and copper(II) acetate (98%; Sigma-Aldrich) were dissolved in approximately 25 mL of distilled water with the addition of a few drops of concentrated HNO_3_. Citric acid (molar ratio metal ions:citric acid = 1:3) was added while stirring. The obtained clear solutions were subsequently dried on a hot plate at 100 °C until gel formation. The gels were further heated at 350 °C until ignition. The resulting powders were calcinated at 950 °C for 6 h. Oxyfluorides La_2_Ni_1–*x*_Cu_*x*_O_3_F_2_ were synthesized from the precursor oxides by mixing with polyvinylidene fluoride powder (PVDF/(CH_2_CF_2_)_*n*_) (Alfa Aesar) in a molar ratio of 1:1 (oxide:CH_2_CF_2_) with a small excess (5%) of the polymer. The mixtures were slowly heated 2 K/min to 350 °C, kept at this temperature for 48 h, and afterward allowed to cool down to room temperature in the box furnace.

### Characterization

X-ray diffraction (XRD) patterns were recorded with Cu-K_α1,2_ radiation on a Bragg–Brentano Bruker D8-Advance diffractometer equipped with a 1D silicon strip detector (LynXeye). Patterns were recorded in the angular range of 2θ = 10–140° with a step size of 0.01° and 3 s per step. Additionally, a STOE STADI-MP transmission geometry diffractometer operating with monochromatic Mo-K_α1_ radiation and equipped with a DECTRIS MYTHEN 1K detector was used to record patterns in the angular range of 2θ = 5–75°.

For Rietveld refinements, GSAS II^24^ was used. The instrumental resolution parameters were obtained from a LaB_6_ (STOE STADI-MP) or Al_2_O_3_ (Bruker D8) reference diffractogram.

The samples were checked for residual polymer by ATR-FT infrared spectroscopy in the range of 4000–250 cm^–1^ with a Bruker Tensor 27 spectrometer.

The La, Ni, and Cu contents of the samples were quantified by X-ray fluorescence spectroscopy (XRF) with a Panalytical Epsilon 4 spectrometer. Data analysis was carried out based on the fundamental parameter approach for pelletized powder samples.

Iodometric titration was used for the determination of the average Ni/Cu oxidation states. About 35 mg of the samples was dissolved in concentrated HCl containing KI in excess. Afterward, 1 g of NaHCO_3_ was added to create a CO_2_-saturated atmosphere, preventing the solutions from autoxidation. For titration, a 0.005 M Na_2_S_2_O_3_ solution was used. The obtained oxidation state was calculated from three averaged titrations per sample.

DFT calculations were performed using the projector augmented wave (PAW) setups^25,26^ as implemented in the Vienna ab initio simulation (VASP V5.4.) package. For structural optimization, uniform Γ-centered k-point grids with a density of 2000 k-points per reciprocal atom were used to sample the Brillouin zones. For calculations of energy differences between magnetic orderings, denser k-grids with a density of 5 k-points/Å^–1^ were applied. A 520 eV cutoff for the plane-wave basis was used, and the total energies were converged to less than 0.01 meV/cell. The Perdew–Burke–Ernzerhof^27^ exchange-correlation functional was used with an on-site 6.2 eV repulsive *U* correction for Ni 3d states. For the compositions containing both Ni and Cu, different magnetic and structural configurations were generated using the cluster expansion method as implemented in the ATAT package.^28^

^19^F magic angle spinning (MAS) NMR experiments were performed on a 7.04 T magnet and a 1.4 T magnet operated with an Avance II Bruker NMR console with Topspin V2.1pl8, operating at frequencies of 286 and 56.4 MHz, respectively. Magic angle sample spinning on the 7.04 T magnet was carried out with a homemade MAS probe head for cylindrical rotors with 4 mm outer diameter spinning at around 8.5 to 10 kHz. The samples were packed in 3D-printed inserts made of polypropylene and using a 3D-printed stator.^29^ Magic angle sample spinning on the 1.4 T magnet was carried out with a homemade conical stator with the sample packed in 3D-printed conical rotors, spinning at approximately 20 kHz.^30^ The chemical shift values refer to neat CFCl_3_.^31^ The spectrum was analyzed with deconv2Dxy.^32^

## Results and Discussion

### Crystal Structure Evaluation of Oxides La_2_Ni_1–*x*_Cu_*x*_O_4_

The oxyfluoride La_2_NiO_3_F_2_ was previously prepared by topochemical fluorination of La_2_NiO_4_ obtained from solid-state synthesis with La_2_O_3_ and NiO as starting materials.^19^ Our own first experiments showed that a successful formation of the desired Cu-containing oxyfluorides was not possible using this approach. Instead, mixtures of the targeted oxyfluorides, LaOF, and unreacted oxides were usually obtained as products.

**Figure 1:**
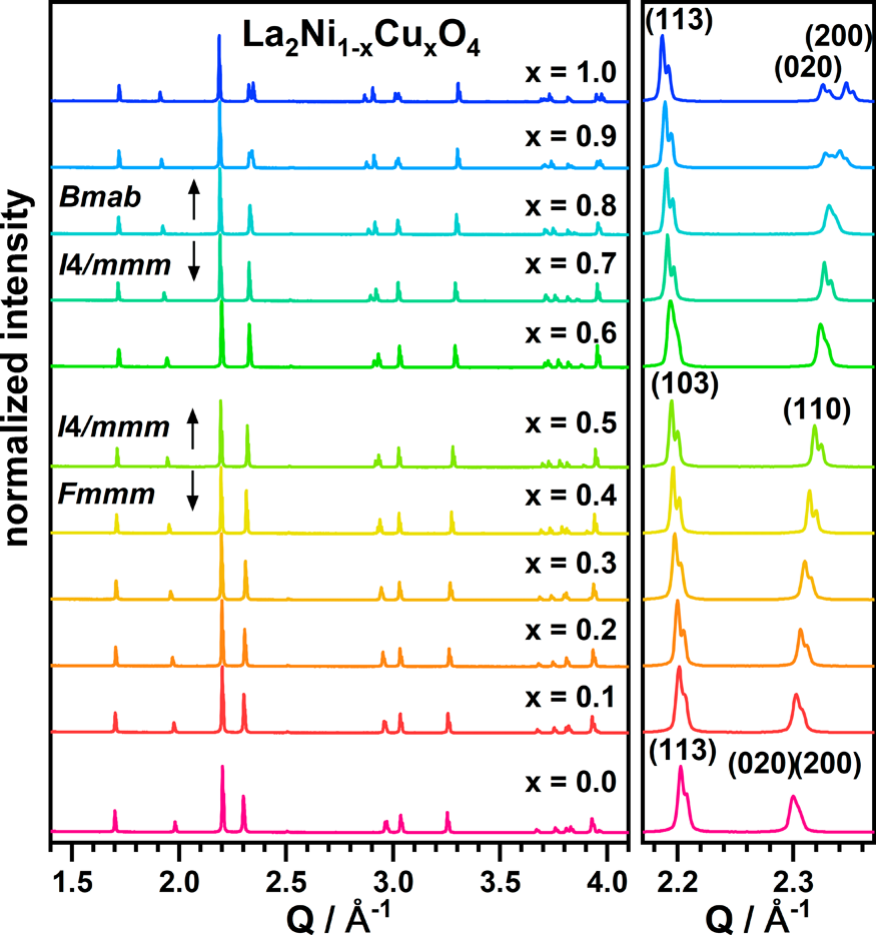
XRD patterns of La_2_Ni_1–*x*_Cu_*x*_O_4_ with an enlarged view of the (103)/(110) main reflections (with respect to the *I*4/*mmm* K_2_NiF_4_ structure) highlighting the splitting of (110) into (200)/(020) for *x* < 0.5, and *x* > 0.7 due to the orthorhombic unit cell symmetry.

The use of more reactive precursor oxides obtained by the citrate-based route at lower calcination temperatures has been successful in the synthesis of La_2_CuO_3_F_2_^20^ as well as La_2_NiO_2.5_F_3_.^33^ Therefore, this approach was used for the precursor oxides in this work. Phase-pure samples of the whole oxide substitution series La_2_Ni_1–*x*_Cu_*x*_O_4_ (Δ*x* = 0.1) were successfully obtained, and their XRD patterns are shown in Figure 1. For substitution levels of *x* ≤ 0.4, an orthorhombic unit cell (*Fmmm*) is indicated by broadening of some peaks with mainly (*hk*0) contribution like the (110) reflection (with respect to the tetragonal (*I*4/*mmm*) K_2_NiF_4_ unit cell). For the range of 0.5 ≤ *x* ≤ 0.7, no such broadening is observed and the compounds possess the tetragonal *I*4/*mmm* unit cell symmetry. For copper contents of *x* ≥ 0.7, again, a clear splitting of the (110) reflection into two separated reflections ((200)/(020)) indicates a transition from the tetragonal (*I*4/*mmm*) to orthorhombic distorted *B*-centered (*Bmab*) unit cell of La_2_CuO_4_. Such phase transitions were previously found by Aguadero et al.^22^ for oxides which were pepared via classical solid-state synthesis with copper contents of *x* = 0.0, 0.2, 0.4, 0.6, and 1. Boehm et al.^21^ reported slightly different structural behavior with an orthorhombic (*Fmmm*)-to-orthorhombic (*Bmab*) transition at *x* > 0.75 but without the presence of a tetragonal structure throughout the substitution series. The structural transitions observed in this work are therefore in good agreement with the already published data yet provide more details because smaller increments of *x* have been used.

**Figure 2:**
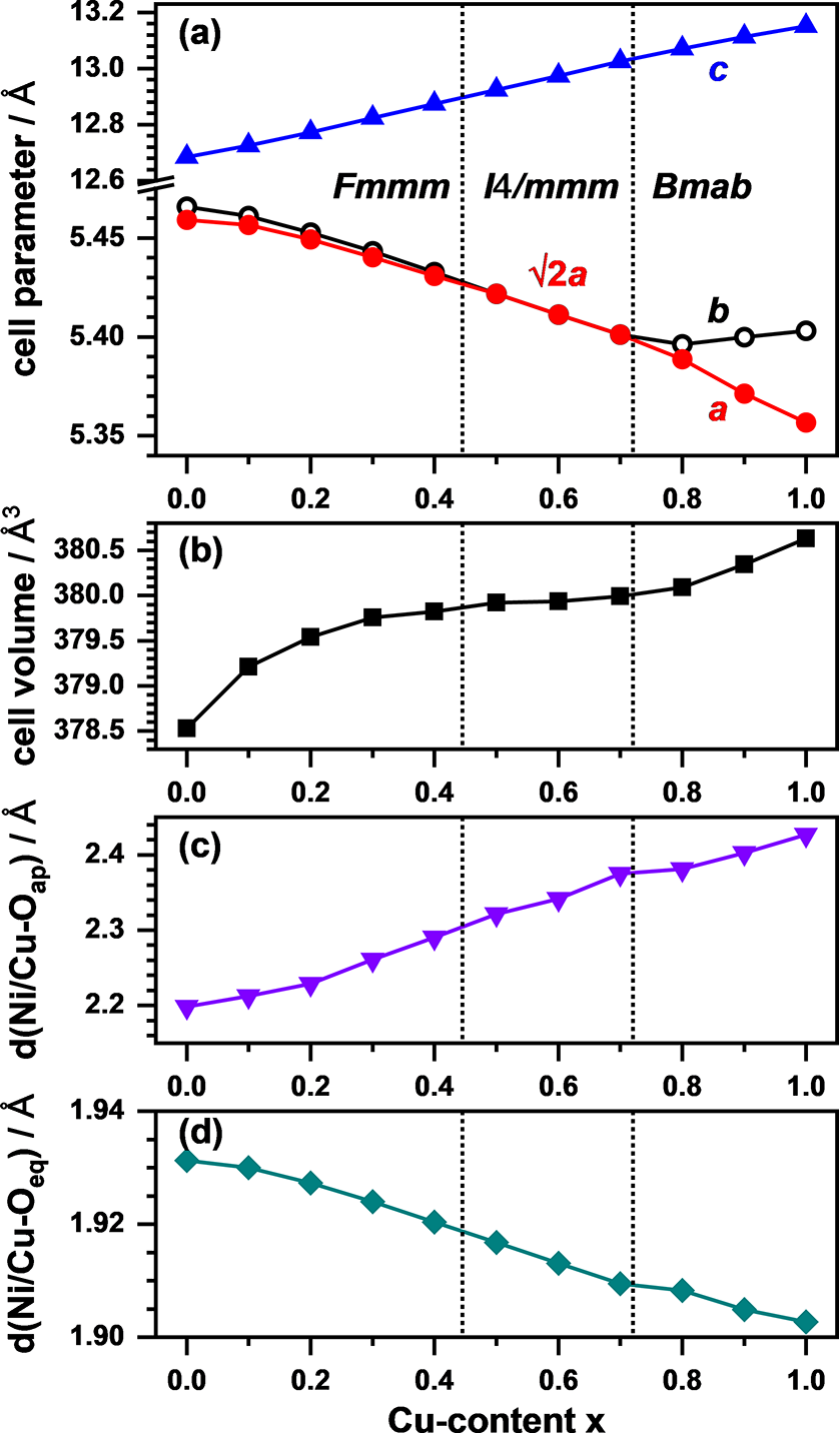
Evolution of the unit cell parameters *a*, *b*, and *c* (a) and cell volume (b) as well as the apical (Ni/Cu–O_ap_) and equatorial (Ni/Cu–O_eq_) distances (c, d) with the copper content (*x*) obtained by Rietveld refinements. The cell parameter *a* is given as √2*a* for *I*4/*mmm* to achieve comparability between the different centered lattices (*Fmmm*, *I*4/*mmm*, and *Bmab*).

Rietveld refinements were applied to study the structural evolution and to obtain unit cell parameters for all samples. The Rietveld plots and all crystallographic parameters are shown in the Supporting Information (Table S1 and Figures S1 and S2). The obtained unit cell parameters are plotted in Figure 2a,b, depending on the Cu content *x*. The increase in unit cell volume is not linear due to an opposed change in parameters *a* and *c*. While *c* increases almost linearly with *x* throughout the whole substitution series, the parameters *a* and *b* decrease with a slightly different slope up to the orthorhombic-to-tetragonal transition at *x* ≥ 0.4. Parameters *a* and *b* are the same for the compounds with 0.5 ≤ *x* ≤ 0.7 due to the tetragonal unit cell symmetry, and they decrease further with a similar slope as before. Above *x* ≥ 0.8, the cell parameter *a* further decreases, while *b* even slightly increases, resulting in a stronger increase in the cell volume.

The overall increase in the unit cell volume is interpreted as the result of an increasing Jahn–Teller distortion of the Ni/CuO_6_ octahedra with *x*. For Ni^2+^ (3d^8^ configuration), no Jahn–Teller distortion is to be expected, while for La_2_CuO_4_, a strong elongation of the octahedra occurs^34,35^ due to the 3d^9^ electron configuration of Cu^2+^. This increasing octahedral distortion is reflected by a strong and almost linear increase in the apical atomic distance *d*(Ni/Cu–O_ap_) from 2.2 to 2.4 Å (*x* = 0.0 to *x* = 1.0) accompanied by a less pronounced decrease in the equatorial atomic distance *d*(Ni/Cu–O_eq_) from 1.93 to 1.90 Å as plotted in Figure 2c,d, respectively.

### Structural and Compositional Characterization of Oxyfluorides La_2_Ni_1–*x*_Cu_*x*_O_3_F_2_

Fluorination of the precursor oxides was carried out with PVDF in the molar ratio of oxide/CH_2_CF_2_ (PVDF) of 1:1. Optimized reaction conditions were established by *in situ* XRD experiments published before for the Cu-rich compounds with *x* = 0.8 and 1.0.^20^ As a result, long dwell times of ∼48 h at a comparatively low reaction temperature of 350 °C were found to give the best results. These optimizations were especially needed for the compounds with higher Cu content, as the thermal stability of the fluorinated phase was found to decrease with increasing Cu content. The corresponding oxyfluorides with the formula La_2_Ni_1–*x*_Cu_*x*_O_3_F_2_ were obtained phase-pure for the entire range of 0 ≤ *x* ≤ 1. The absence of any residual PVDF or other organic phases was confirmed by IR spectroscopy (shown in Figure S3) of the final products. The La:Ni:Cu ratio of both the starting oxides and oxyfluorides was analyzed by XRF measurements. The La:(Ni_1–*x*_Cu_*x*_) ratio was found to be in the range of (1.9–2.1):1, i.e., very close to the expected value, and no major deviations of the nominal nickel/copper ratio were observed. The anionic stoichiometry (3O + 2F) has been verified for both endmembers (*x* = 0.0 and 1.0) lately,^19,20^ and it was also derived from the site occupation factors for the sample with *x* = 0.8 based on neutron powder diffraction data.^20^ As a further support, iodometric titration was applied to determine the bulk oxidation state of nickel/copper in the newly synthesized oxyfluorides. Titration was performed for selected samples (*x* = 0.2, 0.4, 0.6, and 0.8), and the average oxidation state of the B-type cation was found to be near +2 for both the oxides and oxyfluorides without any systematic dependence on the increasing Cu content. These results demonstrate the non-oxidative^36^ nature of fluorination with PVDF and additionally confirm the O_3_F_2_ anion stoichiometry under the assumption of 5 anions per formula.

**Figure 3:**
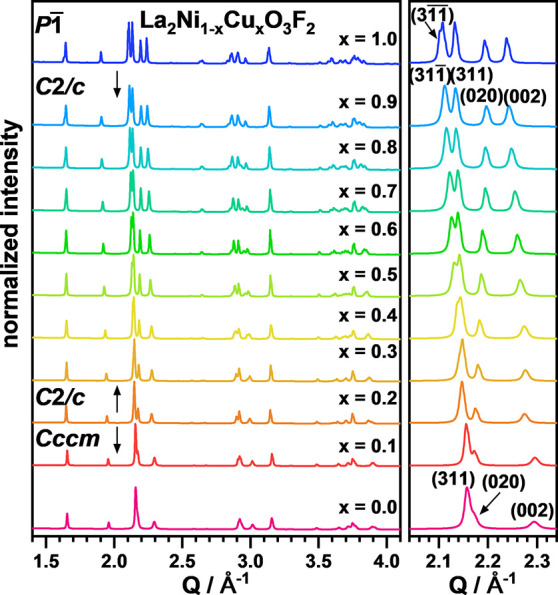
XRD patterns of the oxyfluoride solid solution La_2_Ni_1–*x*_Cu_*x*_O_3_F_2_ with a detailed view of the main reflections (311) and (020)(002), highlighting the position shifts and changing peak splits.

The XRD patterns of the La_2_Ni_1–*x*_Cu_*x*_O_3_F_2_ oxyfluorides are shown in Figure 3. The overall diffraction patterns for *x* ≤ 0.3 resemble the one of La_2_NiO_3_F_2_, even though a significant decrease in the orthorhombic distortion of the unit cell is indicated by a decrease in the (020)(002) splitting. For *x* ≥ 0.4, an additional gradually increasing splitting of the (311) main reflection is observed with increasing *x*, indicating a transition of the orthorhombic structure of La_2_NiO_3_F_2_ (*Cccm*)^19^ to the monoclinic unit cell of La_2_Ni_0.2_CuO_3_F_2_ with *C*2/*c* symmetry^20^ (see Figure S4 for the symmetry relations of *Cccm* and *C*2/*c*). For La_2_CuO_3_F_2_ (*x* = 1), we previously reported additional symmetry lowering to a triclinic structure (*P*-1), which is for example indicated by a shoulder found at the (31-1) reflection (marked with an arrow in Figure 3) arising from the triclinic cell metric.

Rietveld refinements were conducted as joint refinements based on two X-ray diffraction data sets recorded with monochromatic Mo-K_α1_ radiation and Cu-K_α1,2_ radiation. This approach was used to achieve higher redundancy and to combine the advantages of the absence of Ni fluorescence in the Mo-K_α1_ diffraction patterns and their higher accessible *Q*-range with the better angular resolution of the diffraction patterns taken with Cu radiation. In our previous refinements of the *x* = 0.8 compound, a split apical octahedral (X_ap_) position was derived from the refinement of NPD data and this finding was interpreted as the result of a Jahn–Teller distortion of the CuO_4_F_2_ octahedra in contrast to NiO_4_F_2_.^20^ This split position was not considered in the current refinements because the O/F positions cannot be determined with the required accuracy from XRD data, especially not in the presence of heavy scattering atoms like La/Ni/Cu. Rietveld plots of all refinements and the obtained crystallographic parameters can be found in the Supporting Information (Figures S5 and S6 and Table S2). For *x* = 0.0 and 0.1, good refinements were achieved with the structural model of La_2_NiO_3_F_2_ in the space group *Cccm*. For *x* = 0.2 to 0.4, an increasing anisotropic broadening of the (311) reflection indicates the transitions from orthorhombic to monoclinic unit cell symmetry and the space group *C*2/*c* was therefore chosen for all compounds with a copper content of *x* ≥ 0.2. For *x* = 0.9, the monoclinic structural model of the *x* = 0.8 compound and the triclinic structural model of La_2_CuO_3_F_2_ were used for the refinements. For both samples, no systematic broadening or splitting of selected reflections was observed even for the highest *Q* values. Therefore, the monoclinic structure model (*C*2/*c*) was chosen. Additional fits in *P*-1 did not yield significantly better residual values despite the larger number of refinable parameters.

**Figure 4:**
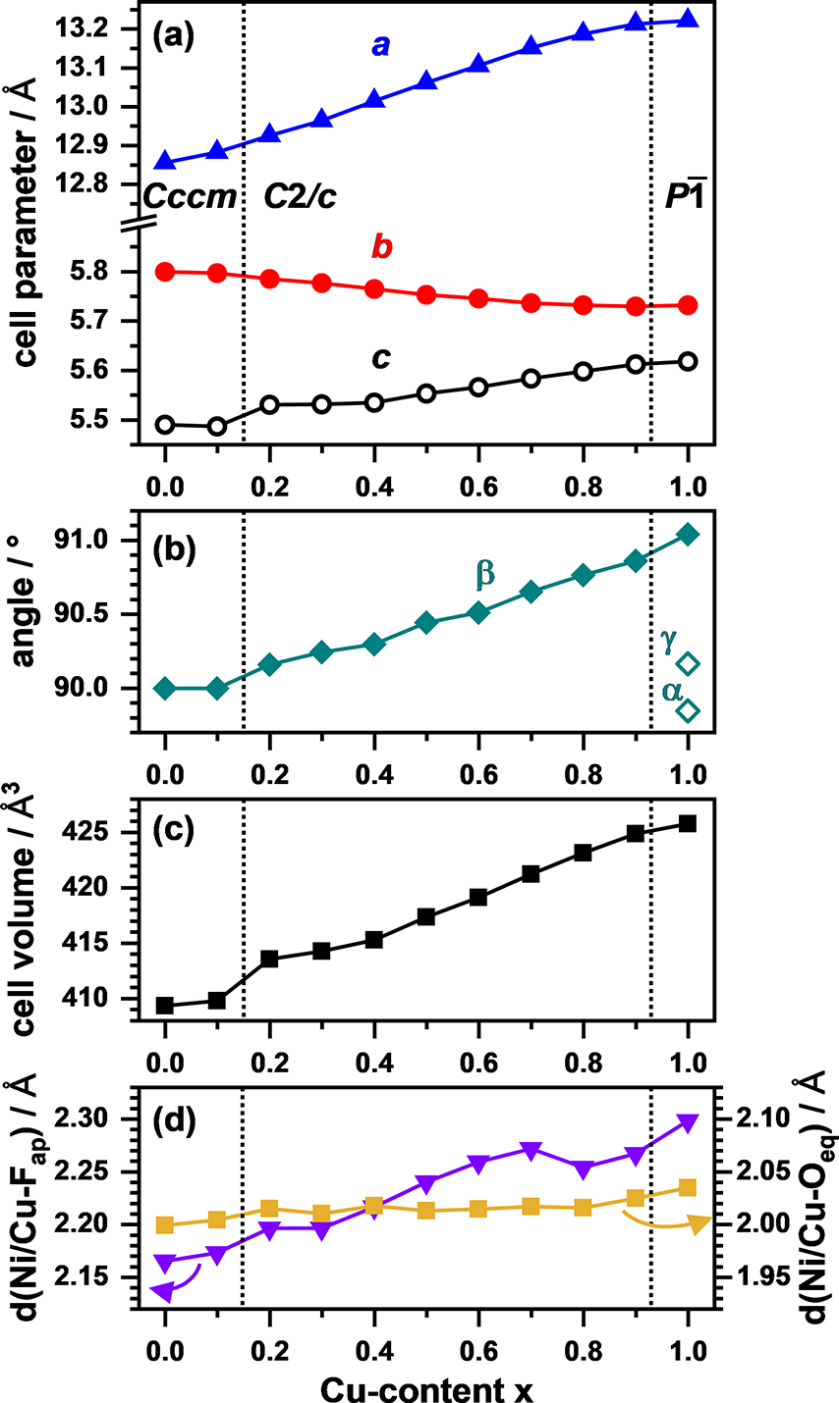
Evolution of the unit cell parameters *a*, *b*, and *c* (a), monoclinic angle β (b), and unit cell volume (c) of La_2_Ni_1–*x*_Cu_*x*_O_3_F_2_ obtained from Rietveld refinements. In panel (d), the evolution of the Ni/Cu–F_ap_ and Ni/Cu–O_eq_ bond distances is shown.

The obtained unit cell parameters are plotted in Figure 4a,b. For the longest axis (*a* in *Cccm* and *C*2/*c*), a strong increase is observed with increasing Cu content ranging from 12.84 Å (*x* = 0.0) to 13.24 Å (*x* = 1.0). This is similar to the observations made for the oxides and can be attributed to the higher space requirements of the Jahn–Teller elongated CuO_4_F_2_ octahedra and the slightly larger ionic radius of Cu^2+^ compared to Ni^2+^ (0.73 Å vs 0.69 Å).^37^ For *x* ≥ 0.7, a reduction in slope is observed, most probably because the elongation is compensated by the increasing octahedral tilting and, in turn, increased monoclinic distortion as reflected by the increase in the angle β. Additionally, the straining of the *b*/*c* plane becomes smaller as *b* decreases and *c* increases with *x*, again being the result of the altered octahedral tilting scheme with additional tilting component in the *c* direction as found for La_2_Ni_0.2_Cu_0.8_O_3_F_2_.^20^ The cell volume (Figure 4c) increases almost linearly with *x* from 410 to 427 Å^3^ despite the observed structural transitions. The Ni/Cu–F_ap_ and Ni/Cu–O_eq_ distances are plotted in Figure 4d, highlighting the impact of substituting Ni^2+^ with the Jahn–Teller active Cu^2+^. As can be seen, the increase in Ni/Cu–F_ap_ with *x* is less pronounced for the oxyfluorides (2.17 Å – 2.30 Å) compared to the one observed for the precursor oxides shown in Figure 2d. This overall reduction in the Ni/Cu–F_ap_ distance is most probably caused by the additional anion in the halite-type layers, which alters the amounts of La–O/F bonds while keeping the overall bond valence sum of +3, resulting in overall increased La–O/F distances, which in return yield a decreased Ni/Cu–F_ap_ distance.

The thermodynamic stability of the oxyfluorides crystallizing in space groups *Cccm* and *C*2/*c* depending on the Ni/Cu ratio was additionally studied by DFT calculations. Thirty-two atom unit cells, with increments of *x* = 0.25, were used to simulate all symmetry-inequivalent Ni/Cu occupational configurations as well as magnetic configurations. The relative energies (Δ*E*) were defined as the difference between the most stable configurations in each phase. The obtained energy difference between *Cccm* and *C*2/*c* can be found in Table 1. The results show that the monoclinic unit cell is lower in energy and, therefore, more stable for all calculated Ni/Cu ratios even for the pure Ni compound La_2_NiO_3_F_2_. This deviation from the experimental results is most probably due to the fact that the energies are calculated for 0 K. Similar results were found by Wissel et al. where the experimental structure of La_2_NiO_3_F_2_ relaxes in a monoclinic unit cell after artificial straining.^38^ The difference in energy between the *Cccm* and *C*2/*c* structures increases with the copper content, which is also reflected by the increasing monoclinic unit cell distortion (i.e. increasing angle β). Based on these findings, the choice of a monoclinic unit cell for the compounds with *x* ≥ 0.2 is justified, although a clear splitting of the diffraction peaks is not in all cases observed due to the limitted resolution of the diffractometers used. To answer the question whether the compounds with *x* < 0.2 also crystallize in a monoclinic unit cell, high-resolution synchrotron XRD data would be needed. Additionally, the smaller value of Δ*E* for *x* = 1 compared to *x* = 0.75 might be seen as a hint to a structural transition in La_2_CuO_3_F_2_, which indeed crystallizes in the space group *P*-1 as reported earlier.^20^ This additional symmetry lowering was not considered by the DFT calculations.

**Table 1:** Energy Difference (Δ*E*) Obtained from DFT Calculations for Both Unit Cell Symmetries (*Cccm* and *C*2/*c*) with Respect to the Copper Content

**copper content (***x*)	Δ*E***(***Cccm*-*C*2/*c***)(meV/atom)**
0	7.76
0.25	8.75
0.5	10.93
0.75	12.11
1	11.42

**Figure 5:**
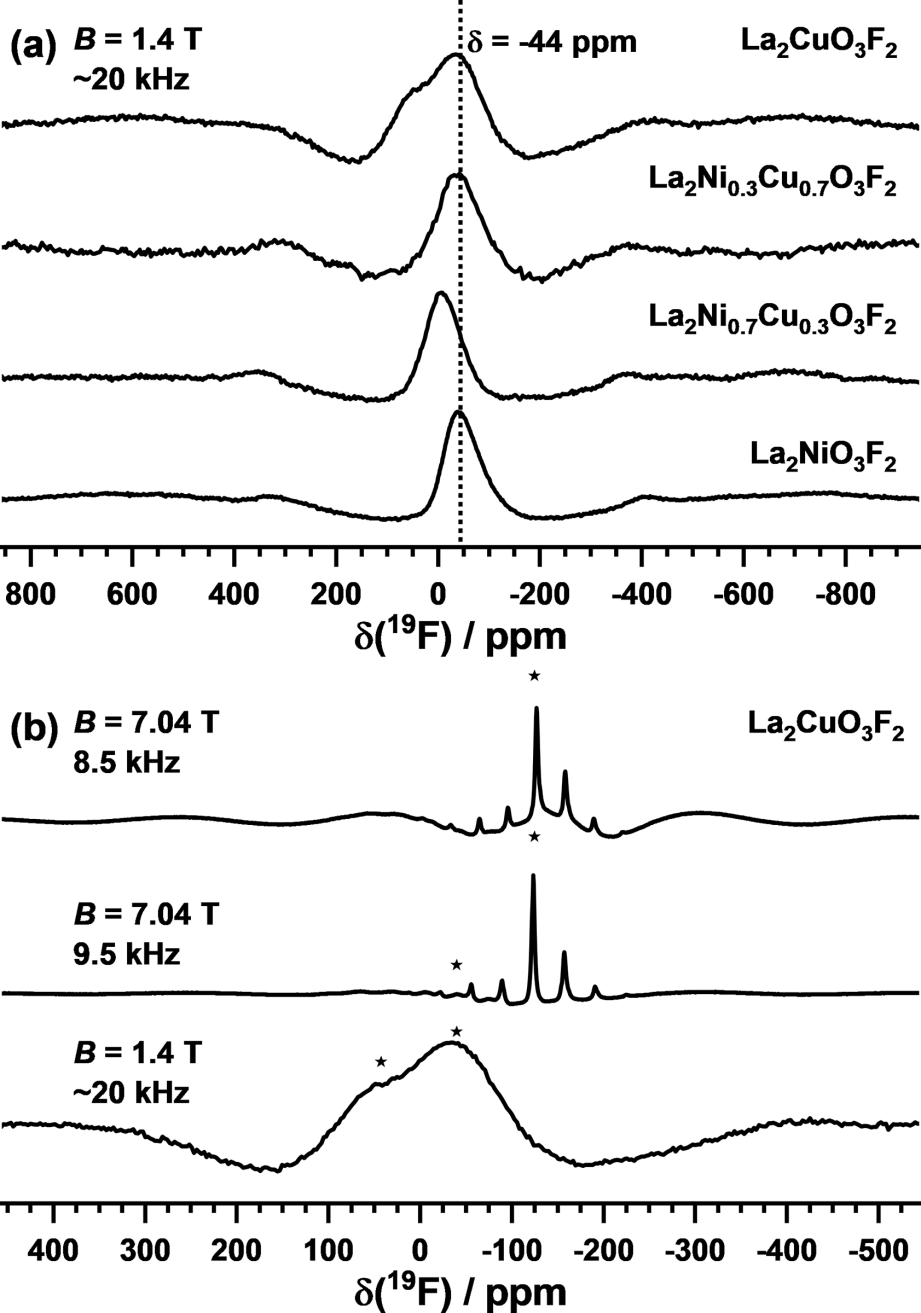
(a) ^19^F MAS NMR spectra of La_2_NiO_3_F_2_, La_2_Ni_0.7_Cu_0.3_O_3_F_2_, La_2_Ni_0.3_Cu_0.7_O_3_F_2_, and La_2_CuO_3_F_2_ measured in *B* = 1.4 T at the spinning frequency of ca. 20 kHz. (b) ^19^F MAS NMR spectra of La_2_CuO_3_F_2_ measured in 7.04 T at spinning frequencies of 8.5 and 9.5 kHz and in 1.4 T at 20 kHz. The asterisks mark isotropic peaks.

^19^F MAS NMR experiments were conducted for both endmembers La_2_NiO_3_F_2_ and La_2_CuO_3_F_2_ as well as two members of the solid solution (*x* = 0.3 and 0.7) to check the previously reported anion ordering. For example, in the case of La_2_NiO_3_F_2_, a single signal at δ = −44 ppm was previously attributed to an apical ordered fluorine atom.^19^ One signal with chemical shifts of δ = −20 ppm (*x* = 0.3) and δ = −44 ppm (*x* = 0.0 and 0.7) is also found in our ^19^F MAS NMR data (see Figure 5a). A possible explanation for the different chemical shifts may be varying La–F interactions due to changes in the chemical environment of fluorine indirectly caused by Ni/Cu substitution and the corresponding Jahn–Teller elongation. The proposed apical F-ordering is in agreement with the previously reported chemical shift values and our observed spectra. Additional NMR data of La_2_CuO_3_F_2_ were recorded on two spectrometers with a 7.04 T magnet and a 1.4 T magnet (Figure 5b). The spectra obtained for the La_2_CuO_3_F_2_ sample on the 7.04 T NMR spectrometer are dominated by spinning side bands of the most intense peak at −127 ppm. By using a 1.4 T magnet in combination with a homemade MAS sample holder,^30^ the spinning side bands were successfully suppressed through high spinning frequencies of ∼20 kHz, yielding two overlapping and thus not well-resolved peaks for La_2_CuO_3_F_2_ centered at 40 and −36 ppm. The peak at −127 ppm of the 7.04 T data is not observed. This signal is attributed to an unknown diamagnetic impurity, which due to the longer relaxation time *T*_1_ of ∼3 s is not observed in the lower field data. This signal is not caused by LaOF for which signals are found at −24 ppm (β-LaOF) or −35 ppm (t-LaOF).^39^ The presence of two signals for La_2_CuO_3_F_2_ is expected as two crystallographic sites (both with Wyckoff symbol 2*e*) exist for the apical position in the previously reported triclinic structure. As the sites share the same multiplicity, a peak area ratio of 50:50 is expected. This ratio is close to the experimental data, which show a ratio of ∼40:60, given that the broad peaks are not fully resolved. Also, the indication of a slight depopulation of one of the apical octahedral positions is possible, probably due to starting decomposition. This is in accordance with the small amounts of LaOF found in the XRD data of this sample.

## Conclusions

The topochemical fluorination of the solid solution La_2_Ni_1–*x*_Cu_*x*_O_4_ was carried out with PVDF as a fluorination agent, yielding phase-pure oxyfluoride samples for the whole substitution series La_2_Ni_1–*x*_Cu_*x*_O_3_F_2_. The structure of the obtained oxides was studied in increments of *x* = 0.1 by X-ray powder diffraction experiments, and two structural transitions were observed (orthorhombic (*Fmmm*; *x* ≤ 0.4) → tetragonal (*I*4/*mmm*; 0.4 *< x* ≤ 0.7) → orthorhombic (*Bmab*; 0.7 *< x* ≤ 1.0)). For the oxyfluorides, we were able to show that the solid solution exhibits a structural transition at *x* = 0.2 from the orthorhombic structure of La_2_NiO_3_F_2_ to the monoclinic distorted structure of La_2_Ni_0.2_Cu_0.8_O_3_F_2_, which we previously solved based on NPD data,^20^ while for La_2_CuO_3_F_2_, even further symmetry lowering to *P*-1 was found. This transition is driven by an increased Jahn–Teller distortion. The structural transition was also studied by DFT calculations, and based on the difference in thermodynamic stability between both space groups, the presence of the monoclinic structure was further confirmed. The previously proposed apical fluoride anion ordering of La_2_NiO_3_F_2_ and La_2_CuO_3_F_2_ was further verified for *x* = 0.0, 0.3, 0.7, and 1.0 by additional ^19^F MAS NMR experiments. In this paper, we were therefore able to show the existence of several new oxyfluorides all exhibiting a similar anion ordering scheme and to refine their strucutres. In a follow-up article, we will discuss the impact of Ni/Cu substitution on the thermal stability as well as the magnetic properties of La_2_Ni_1–*x*_Cu_*x*_O_3_F_2_ as these results would by far exceed the scope of this article.

## Supporting Information Available

FTIR spectra of La_2_Ni_1–*x*_Cu_*x*_O_4_ and La_2_Ni_1–*x*_Cu_*x*_O_3_F_2_ in comparison to PVDF; Rietveld plots from the refinement of XRD data of La_2_Ni_1–*x*_Cu_*x*_O_4_ and La_2_Ni_1–*x*_Cu_*x*_O_3_F_2_; crystallographic data of La_2_Ni_1–*x*_Cu_*x*_O_4_ and La_2_Ni_1–*x*_Cu_*x*_O_3_F_2_ obtained from Rietveld refinements (PDF)
