# Supplementary material for: Ruddlesden–Popper Oxyfluorides La2Ni1–xCuxO3F2 (0 ≤ x ≤ 1): Impact of the Ni/Cu Ratio on the Structure
Source: Inorg Chem. 2024 Mar 20;63(13):6075–81. doi: 10.1021/acs.inorgchem.4c00399 (PMC10988547; doi:10.1021/acs.inorgchem.4c00399)
Supplement: Supplementary file 1 — ic4c00399_si_001.pdf [file ic4c00399_si_001.pdf]

The Ruddlesden-Popper Oxyfluorides $La_2Ni_{1-x}Cu_xO_3F_2$ ($0 \leq x \leq 1$): Impact of the Ni/Cu Ratio on the Structure

Jonas Jacobs[*, †], *Hai-Chen Wang*[‡], *Miguel A. L. Marques*[‡], *Ke Xu*[#], *Jörn Schmedt auf der Günne*[#] *and Stefan G. Ebbinghaus* [†]

[†] Martin Luther University Halle-Wittenberg, Faculty of Natural Sciences II, Institute of Chemistry, Inorganic Chemistry, Kurt-Mothes-Straße 2, D-06120, Halle, Germany

[‡] Research Center Future Energy Materials and Systems of the University Alliance Ruhr, Faculty of Mechanical Engineering, Ruhr University Bochum, Universitätsstraße 150, D-44801 Bochum, Germany

University of Siegen, Faculty IV: School of Science and Technology, Department of Chemistry and Biology, Inorganic Materials Chemistry, Adolf-Reichwein-Str. 2, D-57076, Siegen, Germany

Email: jonas.jacobs@chemie.uni-halle.de

Supporting Information

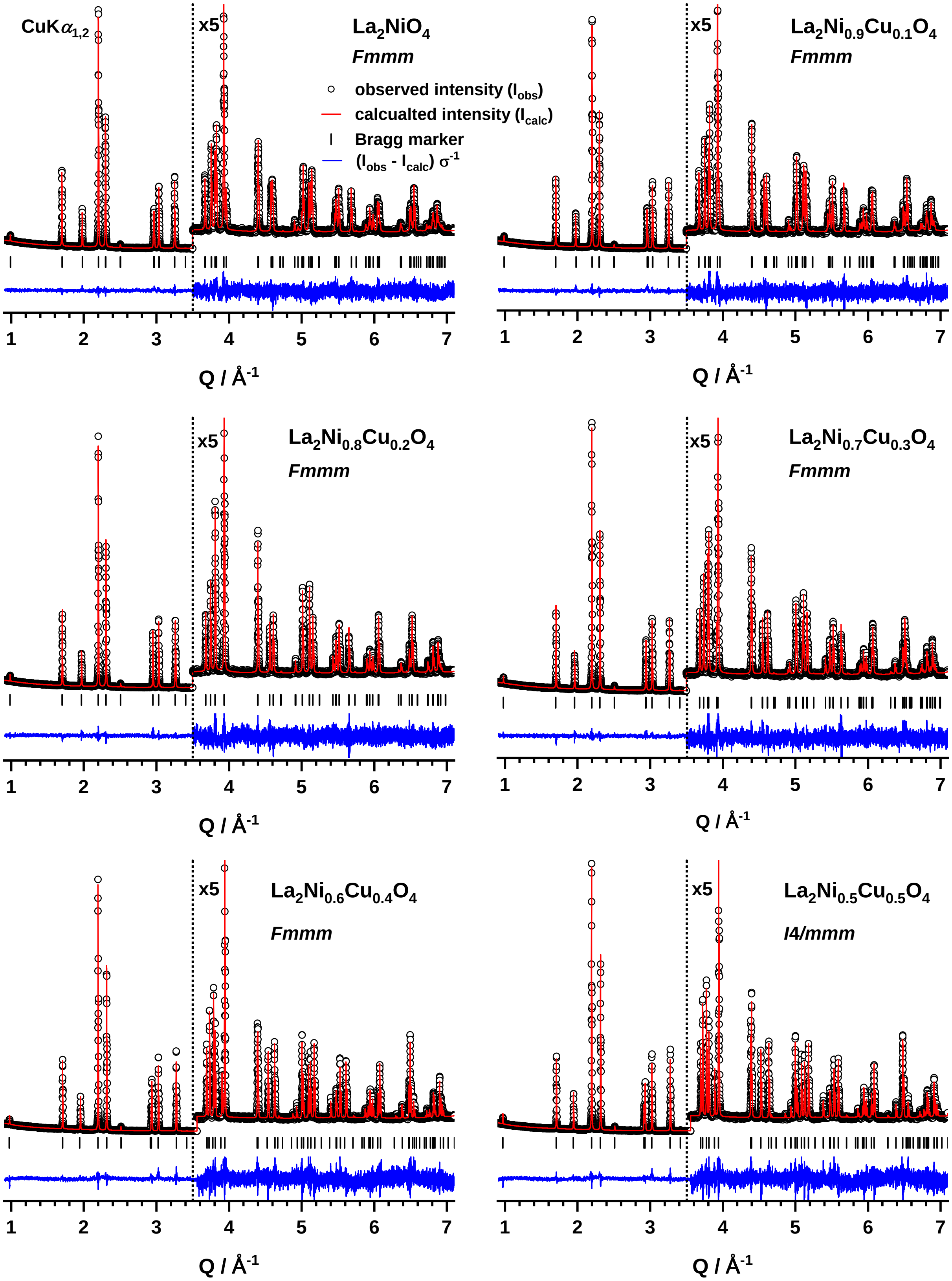

Figure S1: Rietveld plots from the refinements of CuK*α1,2* XRD data for the oxide substitution series $La_2Ni_{1-x}Cu_xO_4$ with $x = 0.0 – 0.5$.

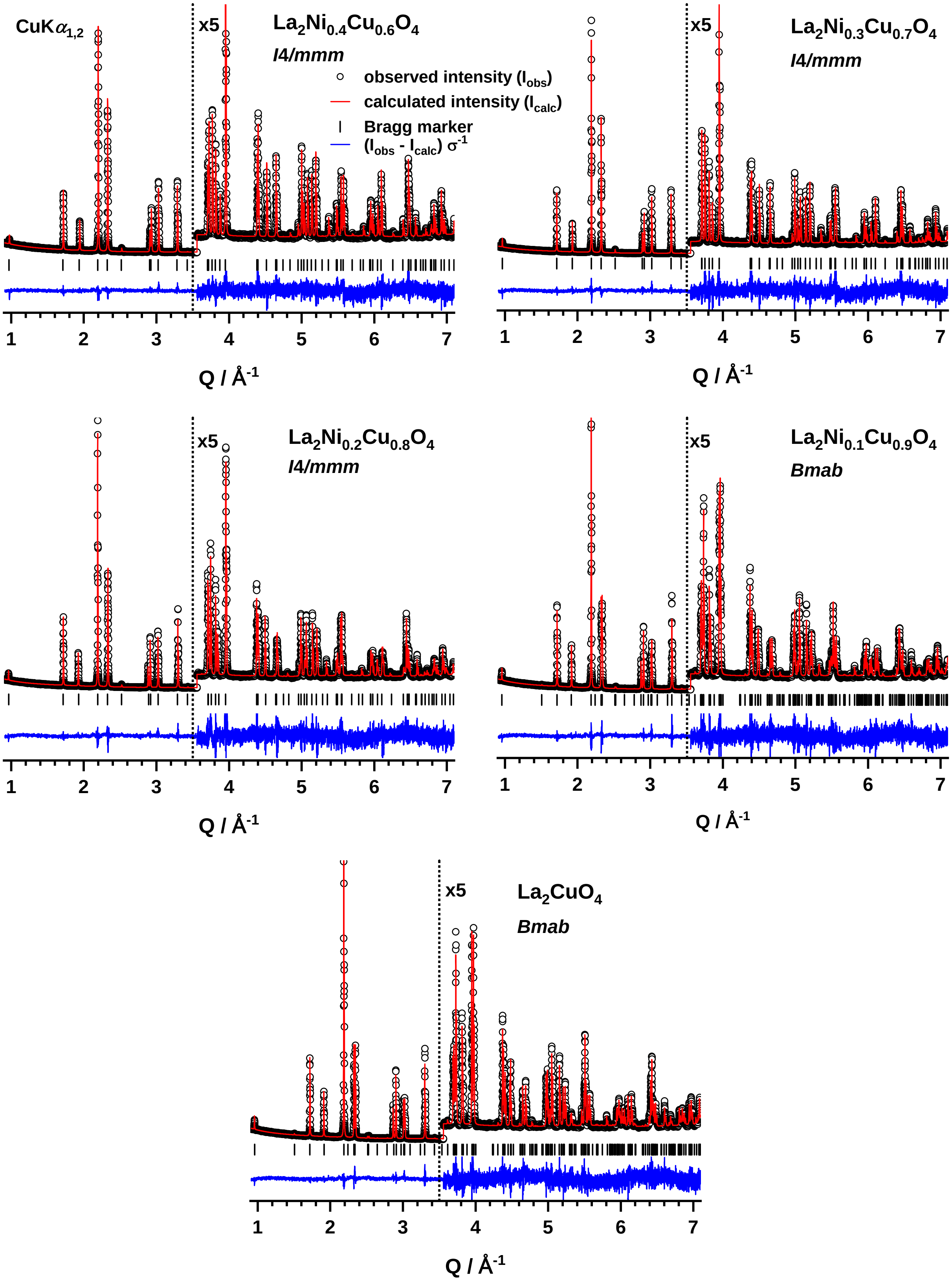

Figure S2: Rietveld plots from the refinements of CuK*α1,2* XRD data for the oxide substitution series $La_2Ni_{1-x}Cu_xO_4$ with $x = 0.6 – 1.0$.

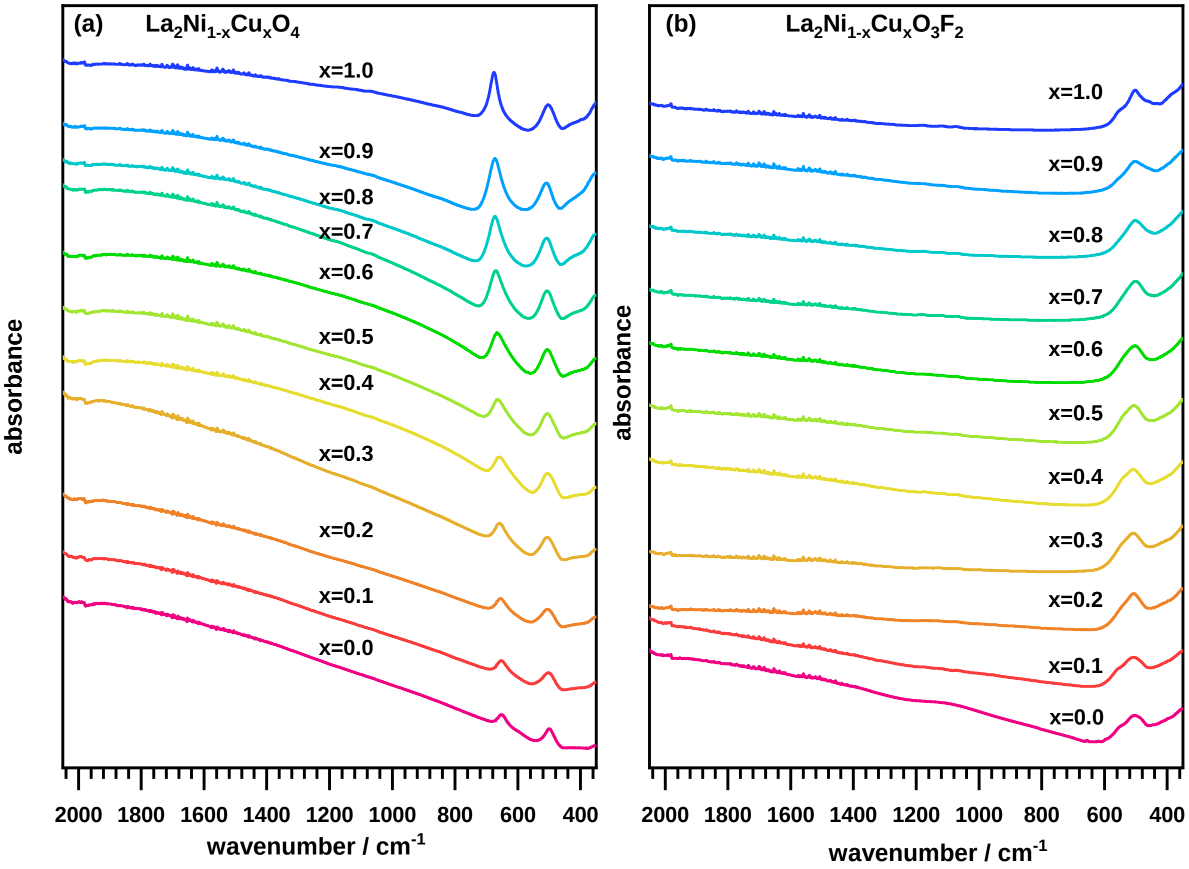

Figure S3: ATR-IR spectra of the oxides $La_2Ni_{1-x}Cu_xO_4$ (a) and oxyfluorides $La_2Ni_{1-x}Cu_xO_3F_2$ (b) of the whole substitution series.

Orthorhombic
***Cccm* (66)**
$a_1 \approx 13.0$
$b_1 \approx 5.75$
$c_1 \approx 5.53$

La: *8l*	Ni: *4e*	$X1_{eq}$: *8g*	$X2_{ap}$: *8l*	$X3_{int}$: *4b*
x	¼	x	x	0
y	¼	0	y	1/2
0	0	¼	0	1/4
x ≈ 0.39		x ≈ 0.25	x ≈ 0.58	
y ≈ 0.75			y ≈ 0.65	

t2 ↓ $a_1 \approx a_2$, $b_1 \approx b_2$, $c_1 \approx c_2$

monoclinic
***C*12/*c*1 (15)**
$a_2 \approx 13.0$
$b_2 \approx 5.75$
$c_2 \approx 5.53$
$\beta \approx 90.5$

La: *8l*	Ni: *4c*	$X1_{eq}$: *8f*	$X2_{ap}$: *8f*	$X3_{int}$: *4e*
x	¼	x	x	0
y	¼	y	y	y
z	0	z	z	1/4
x ≈ 0.39		x ≈ 0.25	x ≈ 0.58	
y ≈ 0.75		y ≈ 0	y ≈ 0.65	y ≈ 0.5
z ≈ 0		z ≈ 0.25	z ≈ 0	

Figure S4: Symmetry tree describing the symmetry reduction from orthorhombic *Cccm* to monoclinic *C*2/*c*. The Anion sites are indicated with an *X* and the subscript indicating equatorial (eq), apical (ap) and interstitial (int) positions.

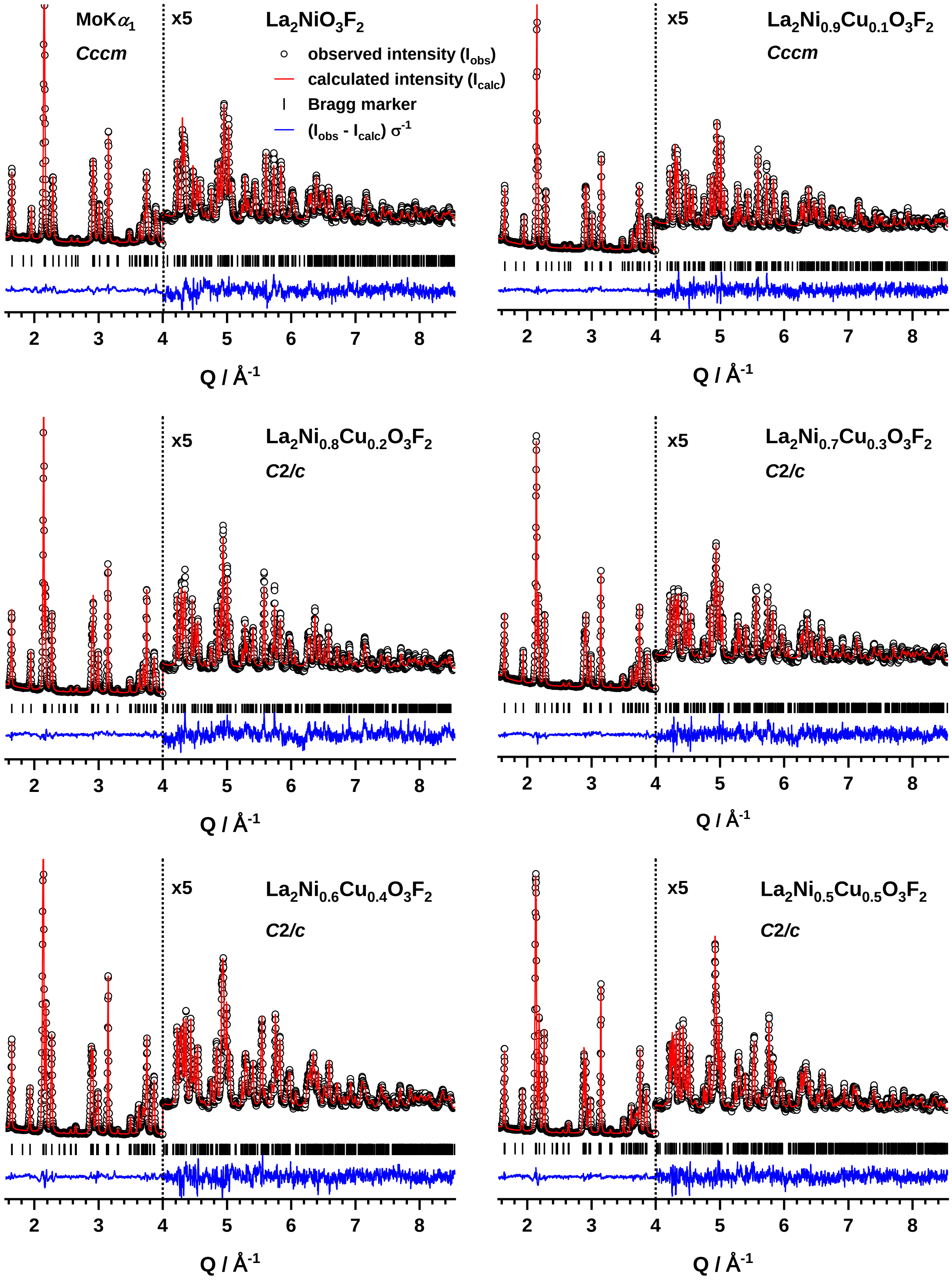

Figure S5: Rietveld plots from the refinements of MoK*α1* XRD data for the oxyfluoride substitution series $La_2Ni_{1-x}Cu_xO_3F_2$ with x = 0.0 – 0.5.

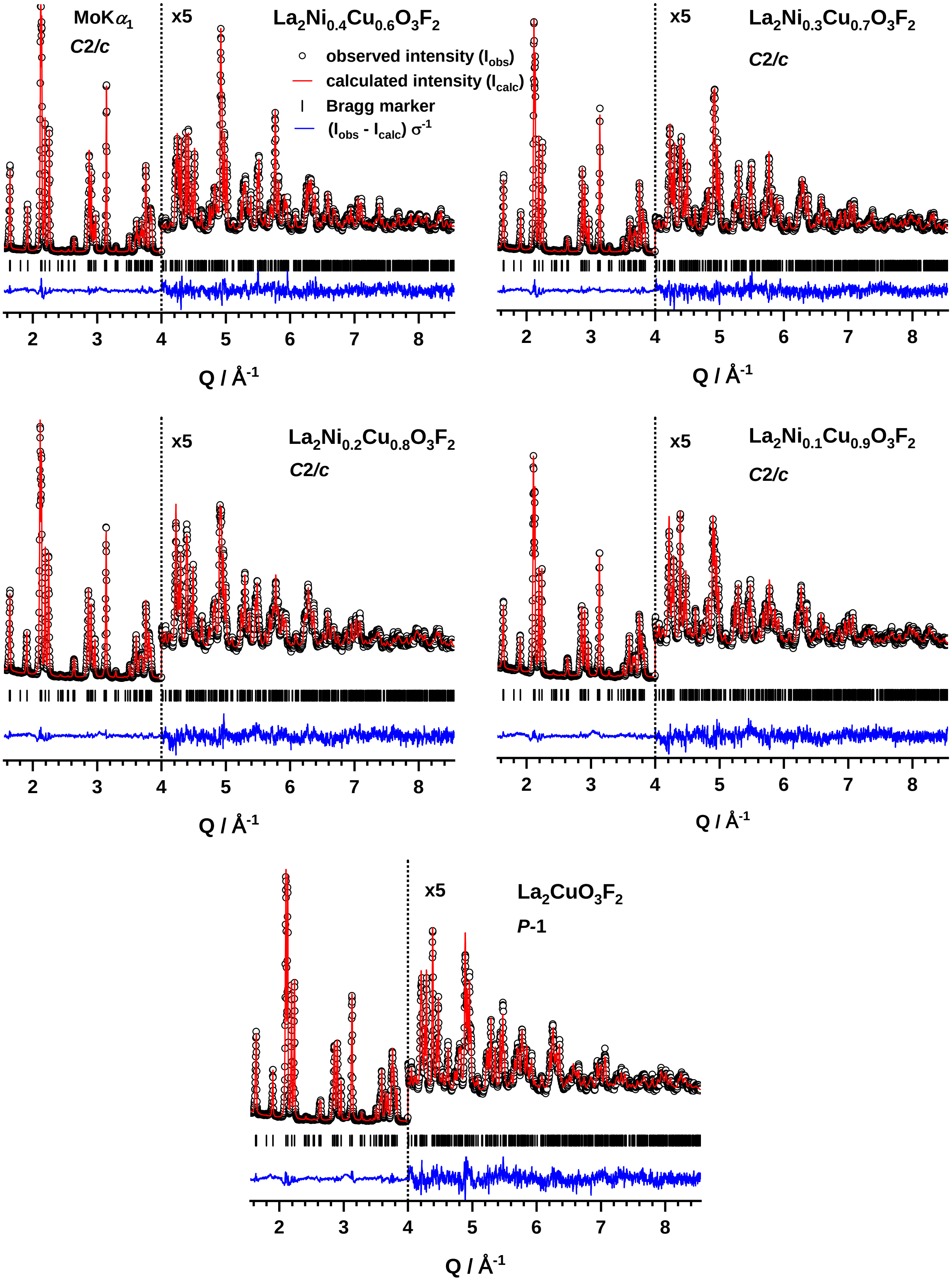

Figure S6: Rietveld plots from the refinements of MoK$\alpha 1$ XRD data for the oxyfluoride substitution series $La_2Ni_{1-x}Cu_xO_3F_2$ with x = 0.6 – 1.0.

Table S1: Structural parameters for the oxide substitution series $La_2Ni_{1-x}Cu_xO_4$ obtained by coupled Rietveld refinements of two independent X-ray (Mo K*α₁* and Cu K*α₁,₂*) diffraction data sets per sample. All positions were assumed to be fully occupied and the theoretical Ni/Cu ratio was used for refinement.

x **in $La_2Ni_{1-x}Cu_xO_4$**		**0.0**	**0.1**	**0.2**	**0.3**	**0.4**	**0.5**	**0.6**	**0.7**	**0.8**	**0.9**	**1.0**
**Space group symbol**		*Fmmm*	*Fmmm*	*Fmmm*	*Fmmm*	*Fmmm*	*I4/mmm*	*I4/mmm*	*I4/mmm*	*Bmab*	*Bmab*	*Bmab*
**a / Å**		5.4593(3)	5.4566(2)	5.4494(4)	5.4402(2)	5.4308(1)	3.8338(2)	3.8264(3)	3.8192(3)	5.3888(2)	5.3713(1)	5.3567(2)
**b / Å**		5.4658(2)	5.4611(5)	5.4529(2)	5.4435(3)	5.4327(2)				5.3962(1)	5.4000(3)	5.4032(2)
**c / Å**		12.6855(1)	12.7256(3)	12.7729(2)	12.8239(4)	12.8737(2)	12.9243(1)	12.9747(2)	13.0256(1)	13.0710(1)	13.1131(1)	13.1509(0)
**Vol / $Å^3$**		378.5(1)	379.2(2)	379.6(1)	379.8(6)	379.8(2)	189.96(1)	189.97(3)	190.00(1)	380.09(2)	380.34(1)	380.63(2)
**La**	**Wyckoff**	***8i***	***8i***	***8i***	***8i***	***8i***	***4e***	***4e***	***4e***	***8f***	***8f***	***8f***
	**x / a**	0	0	0	0	0	0	0	0	0	0	0
	**y / b**	0	0	0	0	0	0	0	0	-0.0011(7)	-0.0059(3)	-0.0074(2)
	**z / c**	0.3609(1)	0.3609(4)	0.3608(4)	0.3609(4)	0.3610(3)	0.3610(3)	0.3611(3)	0.3612(3)	0.3614(3)	0.3615(4)	0.3615(1)
	**Uiso / $Å^2$**	0.0078(1)	0.0103(1)	0.0061(1)	0.0087(1)	0.0077(1)	0.0081(1)	0.0050(1)	0.0067(1)	0.0053(1)	0.0040(1)	0.0028(1)
**Ni/Cu**	**Wyckoff**	***4a***	***4a***	***4a***	***4a***	***4a***	***2a***	***2a***	***2a***	***4a***	***4a***	***4a***
	**x / a**	0	0	0	0	0	0	0	0	0	0	0
	**y / b**	0	0	0	0	0	0	0	0	0	0	0
	**z / c**	0	0	0	0	0	0	0	0	0	0	0
	**Uiso / $Å^2$**	0.0068(3)	0.0088(3)	0.0301(3)	0.0059(3)	0.0010(3)	0.0040	0.0040	0.0040	0.0040	0.0040	0.0040
**O1@X1 (equatorial)**	**Wyckoff**	***4c***	***4c***	***4c***	***4c***	***4c***	***4c***	***4c***	***4c***	***8e***	***8e***	***8e***
	**x / a**	¼	¼	¼	¼	¼	0	0	0	¼	¼	¼
	**y / b**	¼	¼	¼	¼	¼	½	½	½	¼	¼	¼
	**z / c**	0	0	0	0	0	0	0	0	-0.0046(3)	-0.0025(17)	-0.0024(13)
	**Uiso / $Å^2$**	0.0103(9)	0.0105(1)	0.0067(9)	0.0098(8)	0.0074(9)	0.0400	0.0400	0.0400	0.0400	0.0400	0.0400
**O2@X2 (apical)**	**Wyckoff**	***4e***	***4e***	***4e***	***4e***	***4e***	***4e***	***4e***	***4e***	***8f***	***8f***	***8f***
	**x / a**	0	0	0	0	0	0	0	0	0	0	0
	**y / b**	0	0	0	0	0	0	0	0	0.0339(13)	0.0194(24)	0.0203(19)
	**z / c**	0.1733(3)	0.1743(3)	0.1800(4)	0.1771(3)	0.1786(3)	0.1806(3)	0.1804(3)	0.1820(3)	0.1818a(3)	0.1831(3)	0.1845(3)
	**Uiso / $Å^2$**	0.0290(2)	0.0318(2)	0.0326(1)	0.0359(2)	0.0295(1)	0.0400	0.0400	0.0400	0.0400	0.0400	0.0400
**R_W / %**		3.83	4.40	4.18	4.47	4.47	5.007	5.078	5.168	5.679	6.253	5.533
**χ^2**		1.67	2.16	1.93	2.15	1.95	2.55	2.31	2.49	2.93	3.45	2.69
**GOF**		1.29	1.47	1.39	1.47	1.40	1.60	1.52	1.58	1.71	1.86	1.62

Table S2: Structural parameters for the oxyfluorides substitution series $La_2Ni_{1-x}Cu_xO_3F_2$ obtained by coupled Rietveld refinements of two independent X-ray (Mo Kα_1 and Cu K$\alpha_{1,2}$) diffraction data sets per sample. All positions were assumed to be fully occupied and the theoretical Ni/Cu ratio was used for refinement. The parameters of *x* = 1.0 are not shown as they are already published [1].

x in $La_2Ni_{1-x}Cu_xO_3F_2$		0.0	0.1	0.2	0.3	0.4	0.5	0.6	0.7	0.8	0.9
Space group symbol		*Cccm*	*Cccm*	*C2/c*	*C2/c*	*C2/c*	*C2/c*	*C2/c*	*C2/c*	*C2/c*	*C2/c*
a / Å		12.8562(3)	12.8828(2)	12.9266(2)	12.9690(2)	13.0149(3)	13.0631(2)	13.1065(2)	13.1527(2)	13.1873(3)	13.2079(4)
b / Å		5.7995(1)	5.7972(1)	5.7845(1)	5.7767(1)	5.7649(1)	5.7537(1)	5.7457(1)	5.7366(1)	5.7323(1)	5.7274(2)
c / Å		5.4902(1)	5.4874(1)	5.5317(1)	5.5324(1)	5.5350(1)	5.5539(1)	5.5665(1)	5.5839(1)	5.5984(1)	5.6107(2)
β °		90	90	90.14(2)	90.23(2)	90.29(1)	90.44(1)	90.51(1)	90.65(1)	90.76(1)	90.86(1)
Vol / Å^3		409.34(7)	409.82(2)	413.62(3)	414.47(1)	415.52(1)	417.43(2)	419.17(5)	421.28(7)	423.16(4)	424.88(6)
La	Wyckoff	*8l*	*8l*	*8f*	*8f*	*8f*	*8f*	*8f*	*8f*	*8f*	*8f*
	x / a	0.3883(4)	0.3882(3)	0.3882(4)	0.3883(1)	0.3881(4)	0.3887(4)	0.3891(4)	0.3895(4)	0.3901(5)	0.3906(6)
	y / b	0.7462(3)	0.7464(2)	0.7492(3)	0.7503(3)	0.7520(2)	0.7552(2)	0.7594(1)	0.7635(1)	0.7664(1)	0.7693(2)
	z / c	0	0	-0.0056(3)	-0.0074(2)	-0.0098(2)	-0.0109(2)	-0.0130(1)	-0.0150(11)	-0.0159(1)	-0.0175(2)
	Uiso / Å^2	0.0148(1)	0.0149(1)	0.0217(1)	0.0195(1)	0.0164(1)	0.0204(1)	0.0190(1)	0.0166(1)	0.0174(1)	0.0145(2)
Ni/Cu	Wyckoff	*4e*	*4e*	*4c*	*4c*	*4c*	*4c*	*4c*	*4c*	*4c*	*4c*
	x / a	1/4	1/4	1/4	1/4	¼	¼	1/4	1/4	1/4	¼
	y / b	1/4	1/4	1/4	1/4	¼	¼	1/4	1/4	1/4	¼
	z / c	0	0	0	0	0	0	0	0	0	0
	Uiso / Å^2	0.0181(5)	0.0167(4)	0.0171(4)	0.0148(4)	0.0110(4)	0.0123(4)	0.0106(3)	0.0084(3)	0.0091(4)	0.0080(5)
O1@X1 (equatorial)	Wyckoff	*8g*	*8g*	*8f*	*8f*	*8f*	*8f*	*8f*	*8f*	*8f*	*8f*
	x / a	0.2585(7)	0.2647(4)	0.2660(4)	0.2659(4)	0.2691(5)	0.2672(4)	0.2682(4)	0.2684(4)	0.2656(5)	0.2682(6)
	y / b	0	0	-0.0155(3)	-0.005(4)	0.0019(3)	0.0026(3)	-0.0070(2)	-0.0089(2)	-0.0112(2)	-0.0181(2)
	z / c	1/4	1/4	0.2350(39	0.242(3)	0.2365(3)	0.2480(3)	0.2434(2)	0.2412(2)	0.2352(2)	0.2262(3)
	Uiso / Å^2	0.01583	0.01583	0.01583	0.01583	0.01583	0.01583	0.01583	0.01583	0.01583	0.01583
F1@X2 (apical)	Wyckoff	*8l*	*8l*	*8f*	*8f*	*8f*	*8f*	*8f*	*8f*	*8f*	*8f*
	x / a	0.5869(4)	0.5868(3)	0.5868(3)	0.5870(3)	0.5832(3)	0.5846(3)	0.5841(3)	0.5840(2)	0.5856(4)	0.5829(5)
	y / b	0.6567(8)	0.6554(6)	0.6505(6)	0.6498(6)	0.6538(6)	0.6504(5)	0.6465(5)	0.6448(5)	0.6458(6)	0.6445(8)
	z / c	0	0	-0.0382(12)	-0.0279(2)	0.0106(2)	-0.0308(1)	-0.0312(9)	-0.0351(9)	-0.0332(1)	-0.0428(15)
	Uiso / Å^2	0.02989	0.02989	0.02989	0.02989	0.02989	0.02989	0.02989	0.02989	0.02989	0.02989
O2@X3 (interstitial)	Wyckoff	*4b*	*4b*	*4e*	*4e*	*4e*	*4e*	*4e*	*4e*	*4e*	*4e*
	x / a	0	0	0	0	0	0	0	0	0	0
	y / b	1/2	1/2	0.489(3)	0.4870(3)	0.5286(3)	0.4846(2)	0.4826(2)	0.04802(2)	0.4844(2)	0.4887(2)
	z / c	1/4	1/4	1/4	1/4	1/4	1/4	1/4	1/4	1/4	1/4
	Uiso / Å^2	0.01583	0.01583	0.01583	0.01583	0.01583	0.01583	0.01583	0.01583	0.01583	0.01583
R_W / %		4.593	4.043	4.426	4.331	4.715	4.491	4.642	4.582	5.152	5.728
χ^2		2.25	1.69	1.94	1.87	2.23	1.93	2.04	1.93	2.32	2.72
GOF		1.50	1.30	1.39	1.37	1.49	1.39	1.43	1.39	1.52	1.65

[1] J. Jacobs et al., Cuprate Oxyfluorides La2Cu0.8Ni0.2O3F2 and La2CuO3F2 with “Channel-like” Anion Ordering. *Inorg. Chem.*, **61** (2022) 17202 (doi:10.1021/acs.inorgchem.2c02776)
